# Comparison of Immune Responses in Pigs Infected with Chinese Highly Pathogenic PRRS Virus Strain HV and North American Strain NADC-20

**DOI:** 10.2174/1874357901711010073

**Published:** 2017-06-30

**Authors:** X. Li, A. Galliher-Beckley, L. Wang, J. Nietfeld, W. Feng, J. Shi

**Affiliations:** 1 Department of Anatomy and Physiology, College of Veterinary Medicine, Kansas State University, Manhattan, KS, USA; 2 Department of Diagnostic Medicine and Pathobiology, College of Veterinary Medicine, Kansas State University, Manhattan, KS, USA; 3 State Key Laboratory of Agrobiotechnology, China Agriculture University, Beijing, China

**Keywords:** HP-PRRSV, HV-PRRSV, NADC-20, Immune responses

## Abstract

**Introduction:**

Chinese HP-PRRSV characterized by high morbidity and mortality of all ages of pigs emerged since 2006 in China. The immune response of HP-PRRSV was never compared with conventional low pathogenic PRRSV strain.

**Objective:**

In this study, we compared the immune responses elicited by a Chinese HP-PRRSV strain HV and a North American RRSV strain NADC20 infections.

**Result:**

Pigs infected with NADC-20 showed significantly higher Ab titers than HV-PRRSV infected pigs at 9 DPI. Infection with HV-PRRSV induced a significantly higher levels of TNF-α and IL-10 in both sera and lung tissues and higher IFN-α and IFN-γ in the serum. Flow cytometry analysis showed that HV-PRRSV infected pigs generated significantly higher frequencies of NK cells in the peripheral blood and Th/memory, CTLs, and T-reg cells in the lung as compared with NADC-20 infected pigs.

**Conclusion:**

This study demonstrates that different immunity profiles were elicited by HV-PRRSV and NADC-20, and these differences may contribute to the distinct pathogenesis of HV-PRRSV and NADC-20.

## 1 INTRODUCTION

Highly-pathogenic PRRS virus (HP-PRRSV) belongs to type 2 genotype (North American, prototype strain VR-2332) of PRRSV, which is a member of the genus *Arterivirus*, family *Arteriviridae*. HP-PRRSV is characterized by high fever and high rates of morbidity and mortality [1]. This novel and highly virulent variant of PRRSV, which first emerged in China in 2006, has rapidly spread to most countries in South East Asia [2]. HP-PRRSV exhibits more tissue tropism than classic PRRSV [3]. Besides lymphoid tissues, IHC examination showed that HP-PRRSV antigen can also be detected in other tissues including trachea, esophagus, liver, kidney, cerebellum, stomach, and intestine [4].

Compared with the prototype of type 2 genotype strain VR-2332, HP-PRRSV can elicit strong immune responses by the evidence of a striking elevation in the level of cytokines associated with both innate and adaptive immunity in HP-PRRSV infected pigs [5]. In contrast, VR-2332, first isolated in 1987, only leads to mild clinical symptoms and does not circulate in the field any longer [6]. NADC-20 is a virulent North American PRRSV strain, which was first isolated in an “atypical PRRSV abortion storm” in 2001 [7]. It has been widely used for viral challenge to evaluate the efficacy of PRRSV vaccines in the U.S [8]. Compared with the other strains of PRRSV in the U.S., NADC-20 can lead to clinical fever (≥ 40°C) and more robust immune responses after infection of pigs [8]. Therefore, analysis of the host immune responses elicited by two virulent strains of PRRSV will contribute to better understanding of the pathogenesis of HP-PRRSV and facilitate more effective vaccine development. In this study, 7-week old pigs were infected with the HV-PRRSV (a HP-PRRSV isolate, GenBank accession no. JX317648), or NADC-20 strain of PRRSV and the clinical symptoms and the profiles of host immune response were compared.

## 2 MATERIALS AND METHODS

### 2.1 Cells and Virus

MARC-145 cells were maintained in modified Eagle’s medium (MEM) supplemented with 7% fetal bovine serum (FBS) containing 100U penicillin/ml and 100ug streptomycin/ml at 37°C with 5% CO_2_. For virus infection and titration, MEM supplemented with 2% FBS was used. HV-PRRSV was rescued from HP-PRRSV infectious clone [9], and propagated on MARC-145 cells for three passages before use. PRRSV NADC-20 was a kind gift from Dr. Lager Kelly (National Animal Disease Center, USDA-ARS, Ames, IA).

### 2.2 Experiment Design

All animal experiments were approved by the Institutional Animal Care and Use Committee at Kansas State University. Briefly, fifteen conventional Large White-Duroc crossbred weaned specific-pathogen free piglets (Female, 7 weeks of age) were tested to be PRRSV negative by ELISA and real time RT-PCR and were divided into 3 groups. Five pigs were infected with NADC-20 (2x10^5^ TCID_50_/pig) and housed for 10 days before the necropsy within the Large Animal Research Center facility (Bio-safety Level 2) at Kansas State University. Another 10 pigs were divided into two groups (n=5/group) and housed in separate rooms within the Biosafety Research Institute (Bio-safety Level 3) at Kansas State University. One group of pigs were infected with HV-PRRV (2x10^5^ TCID_50_/pig) on day 0, and another group of pigs received MEM medium and served as negative controls throughout the study. Weight measurements and blood samples were collected every 3 days and rectal temperature and clinical signs were monitored daily. All pigs were humanely euthanized at 10 DPI. Thymic and lung tissues were weighed and compared with total body weight to evaluate thymic atrophy and lung inflammation induced by the viral infection. Serum samples were used to measure viral load, PRRSV-specific antibodies and cytokine expression [10].

### 2.3 Collection of Blood Samples for Analysis

Blood was collected from each pig at 3, 6, and 9 days post PRRSV infection. Serum was separated from clotted blood and preserved at -20°C. Serum was used for evaluation of viral titer and PRRSV-specific ELISA antibody titers (Herdchek Porcine Reproductive and Respiratory Syndrome Antibody test Kit, IDEXX Laboratories) as previously described [10]. Pig serum and the supernatant of lung homogenates were used to cytokine expression analysis as described earlier [11]. IFN-α and IFN-β ELISA kits were purchased from Abcam (Abcam, Cambridge, MA). IL-4, IL-8, IL-10, IFN-γ and TNF-α were purchased from Invitrogen (Life Technologies, Carlsbad, CA). Procedures were performed as per the manufacturer’s instructions. For a given sample, the OD_450_ was then transformed to concentration by applying a linear regression formula calculated from the results of the standards provided in each kit.

Total RNA was extracted from serum and One-step Taq-Man qPCR was performed to calculate PRRSV RNA copy number in the serum sample according to the brochure of manufacture (EZ-PRRSV^TM^ MPX4.0 Real Time RT-PCR, Tetracore Inc., Rockville, MD). A standard curve was constructed by preparing serial dilutions of an RNA control, supplied in the RT-PCR kit, and virus quantities of unknown samples were determined by linear extrapolation of the Ct value plotted against the standard curve.

Heparinized whole blood was subjected to flow cytometry analysis to determine different lymphocyte populations based on the cell surface marker phenotype: T-helper cells (CD3^+^CD4^+^CD8^-^), cytotoxic T lymphocyte (CD3^+^CD4^-^CD8^+^), Th/memory cells (CD3^+^CD4^+^CD8^+^), T-regulatory cells (CD4^+^FoxP3^+^CD25^+^), NK cells (CD3^-^CD8^+^) and γδ T cells (CD8^+^TcR1N4^+^) as we described earlier [11]. The mouse anti-pig TcR1N4 antibody was purchased from VMRD (Pullman, WA), and all other antibodies were purchased from BD Biosciences (San Jose, CA). Immuno-stained cells were acquired using a FACS Caliber (BD Biosciences) flow cytometer as previously described [11]. Briefly, PBMC was treated with 2% pig serum to block Fc receptors.

Cells were then stained with an appropriate Ab which was either directly conjugated to a specific fluorochrome or with a purified Ab to pig specific immune cell surface marker (TcR1N4). For cells stained with a purified Ab, labeled cells were treated with anti-species isotype specific secondary Ab conjugated with fluorochrome. Finally, cells were fixed with 1% paraformaldehyde before flow cytometer reading. Percentages of each lymphocyte population were analyzed by 100,000 unique events using FlowJo software (Tree Star, Inc., OR, USA).

### 2.4 Histopathological Analysis

Pigs were humanely euthanized at 10 DPI as approved by the Kansas State University Institutional Animal Use and Biosafety Committee. Pathology in the lung was macroscopically and microscopically evaluated and graded as previously described [12]. Briefly, the dorsal and ventral surfaces of each lung lobe were given a score representing the approximate proportion that was consolidated.

 Individual lobe scores were used to determine an overall lung score representing the percentage of lung with macroscopically evident consolidation. Sections of each of the 4 lobes of the right lung were fixed in 10% buffered neutral formalin, paraffin-embedded, sectioned, and stained with hematoxylin and eosin (H&E). Scoring of microscopic lung pathology on a 4 point scale was done in a blinded fashion by a veterinary pathologist in the Kansas State Veterinary Diagnostic Laboratory [12].

### 2.5 Statistical Analysis

All data were expressed as the mean value of five pigs ± SEM. The differences in the level of humoral response, body temperature and body weight, viral titer, lung score, cytokine production, and percentage of lymphocyte subpopulations among each group were determined by the one-way analysis of variance (ANOVA) followed by post-hoc Tukey’s test using Sigmaplot 11 software (Systat Software Inc., San Jose, CA). Differences were considered statistically significant when p<0.05.

## 3 RESULTS

### 3.1 Pigs Infected with HV-PRRSV had Significantly Higher Fever and Less Body Weight Gain as Compared with NADC-20 Infected Pigs

**Fig. (1):**
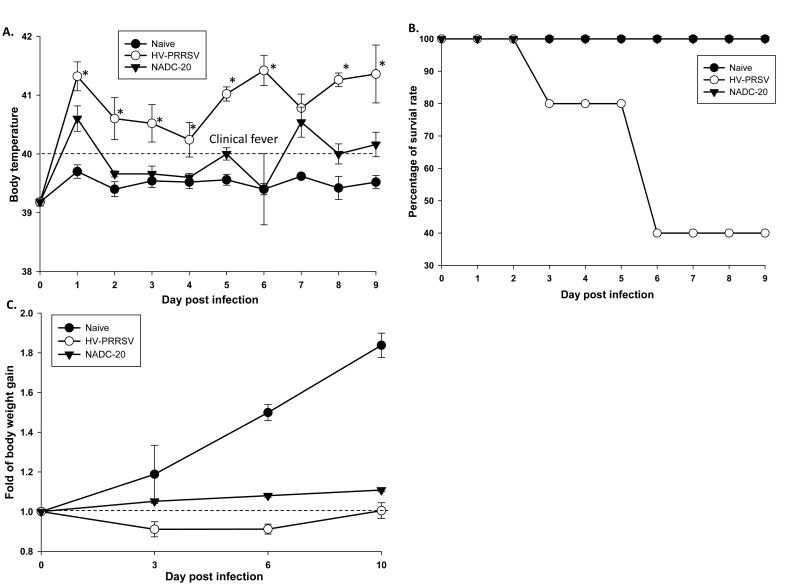
**Fever, mortality rate and loss of body weight after PRRSV infection**. **(A)** Rectal temperatures of all pigs were monitored daily after PRRSV infection. **(B)** Survival rate. **(C)** Fold of total body weight gain during the duration of the experiment was calculated by considering the weight of the pig on day 0 as 1. Each bar represents the average of five pigs ± SEM. *p<0.05.

HP-PRRSV infection is characterized by high fever, high percentage of morbidity and mortality in pigs [1]. Thus, the rectal temperature of pigs was monitored daily. The average body temperature in HV-PRRV infected pigs was above 40°C, the cutoff of clinical fever throughout the study, and it was significantly higher than that in NADC-20 infected pig except at 0 and 7 DPI (Fig. **1A**). The NADC-20 infected pigs developed clinical fever only at 1 and 7 DPI, with the mean body temperature on these two days being 40.5°C.

 One pig within the HV-PRRV infection group died at 3 DPI and two other pigs were euthanized due to severe weakness and moribund condition at 6 DPI (Fig. **1B**). The clinical signs of HV-PRRSV-infected pigs included dehydration, respiratory distress, shivering, and inability to bear weight on front limbs. Two of the dead pigs developed cutaneous hemorrhages and cyanotic extremities on the edges of their ears. None of pigs in the NADC-20 infection group or control group were found dead or moribund to warrant euthanasia. Pigs in the NADC-20 infection group showed transient fever, but no other clinical symptoms were observed. HV-PRRSV infected pigs rapidly lost their body weight when compared with the negative control and NADC-20 infected pigs (Fig. **1C**).

### 3.2 HV-PRRSV Infection Led to Severe Thymus Atrophy and Lung Inflammation in Pigs

**Fig. (2):**
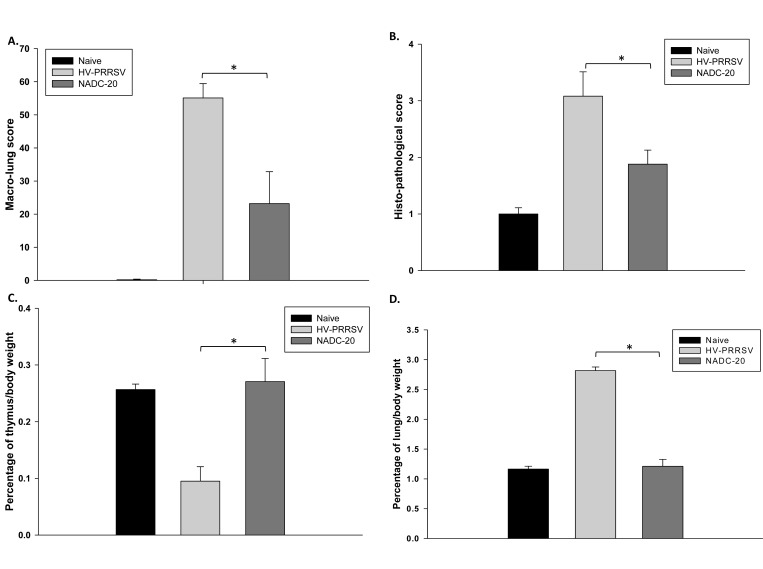
**Lung pathology, thymus atrophy and lung inflammation caused by PRRSV infection.** **(A)** Macro lung pathology score. **(B)** Histopathological score. Thymus weight **(C)** and lung weight **(D)** to body weight ratios after virus infection. Each bar represents the average of five pigs ± SEM. *p<0.05.

Severe lesions, including marked interstitial pneumonia, lymphadenopathy and thymic atrophy were observed in HV-PRRSV infected pigs. Postmortem findings include pulmonary edema, hematoma, pleural adhesion, peritoneal and pericardial effusions, and renal petchia. Pigs in the HV-PRRSV infection group showed more severe and extensive pneumonia than NADC-20 infected pigs, and the macro- and histopathological lung scores in this group were significantly higher than NADC-20 infected group (Fig. **2A**, **B**). No pathologic lesions were identified in control pigs.

HV-PRRSV was previously reported to lead to thymus atrophy [3]. To confirm this, the ratio of thymus/total body weight was calculated to evaluate the thymus atrophy at necropsy. The ratio of thymus/total body weight of pigs in HV-PRRSV infection group was significantly lower as compared with NADC-20 infection group (Fig. **2C**), which supports that severe thymus atrophy occurs in HV-PRRSV infected pigs. In contrast, the thymus weights of pigs infected with NADC-20 showed the similar average weight to that of the negative control pigs. The ratio of lung/total body weight was used to evaluate the inflammation status after viral infection. The ratio was significantly higher in HV-PRRSV infected pigs than that in NADC-20 infected pigs (Fig. **2D**), and there was no difference in the ratio between NADC-20 infected pigs with negative control pigs. The above data showed that HV-PRRSV infection lead to significant thymus atrophy and lung inflammation as compared with NADC-20 infection in pigs.

### 3.3 HV-PRRSV Infection Showed Enhanced Viral Titers in Pigs but did not Elicit Earlier or Higher PRRSV-Specific IDEXX ELISA Antibodies than NADC-20 Infected Pigs

**Fig. (3):**
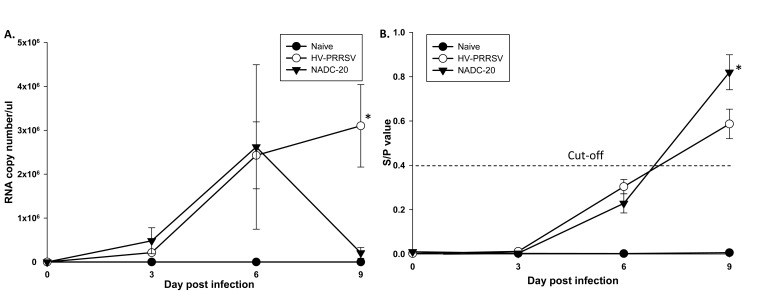
**Viremia and PRRSV-specific ELISA Ab profiles after PRRSV infection.** **(A)** PRRSV viral RNA in the serum was determined by real time RT-PCR. **(B)** Pig serum was assayed for PRRSV-specific antibodies with IDEXX HerdCheck ELISA. The threshold for seroconversion was set at a sample-to-positive (s/p) ratio of 0.4 according to manufacturers’ instructions. Each bar represents the average of five pigs ± SEM. *p<0.05.

HP-PRRSV was previously reported to have higher proliferation ability than the classic PRRSV strains [5]. Indeed, the virus RNA copy number in the serum was higher in HV-PRRSV infected pigs than NADC-20 infected pigs at 3 DPI; however the difference was not significant (Fig. **3A**). At 6 DPI, the viremia in the blood was similar (average 2.5x10^6^ RNA copy number/μl) in both challenge groups. By 9 DPI, the viral titer in NADC-20 infected pigs dropped more than 10 folds, whereas the serum virus copy number of HV-PRRSV infected pigs increased to 3x10^6^ RNA copy number/μl. PRRSV-specific antibodies elicited by the two strains of PRRSV were measured by IDEXX ELISA kit [13]. The high proliferation ability of HP-PRRSV did not elicit earlier or higher titer of PRRSV-specific Ab. At 9 DPI, the average ELISA antibody titer in NADC-20 infected pigs was significantly higher than that in HV-PRRSV infected pigs (Fig. **3B**).

### 3.4 Cytokine Expression in the Serum and Lungs was Up-regulated by HV-PRRSV Infection Compared with NADC-20 Infection

**Fig. (4):**
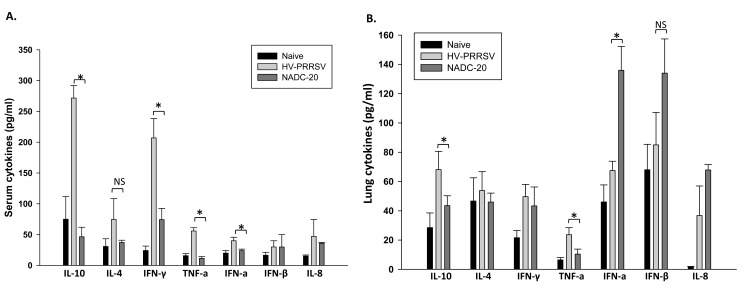
**Serum immune cytokine profiles after PRRSV infection.** Cytokine expression profiles in the sera **(A)** of challenge pigs 6 days post infection (DPI) and supernatants of lung homogenates **(B)** were tested by quantitative ELISA. Data were shown as mean ± SEM for 5 pigs per group. One asterisk denotes a statistically significant difference (P <0.05), and “NS” denotes there was no statistically significant difference.

Sera collected at 6 DPI and the supernatant of lung homogenates collected at 10 DPI were analyzed for innate cytokine (TNF-α, IFN-α, IFN-β, and IL-8) and adaptive cytokine (IL-10, IL-4, and IFN-γ) expression. As for the innate cytokines, HV-PRRSV infection induced significantly higher TNF-α level in both serum and lung samples from the pigs (Figs. **4A**, **B**). HV-PRRSV infection also induced significantly higher IFN-α in the serum but significantly lower IFN-α in the lung samples as compared with NADC-20 infected pigs. There was no significant difference between the two infected groups for the expression of IFN-β and IL-8 in serum and lung samples. As for the adaptive cytokines, HV-PRRSV infection elicited significantly higher IL-10 and IFN-γ in the serum of pigs, and significantly higher IL-10 in the lung samples sample as compared with NADC-20 infected pigs Fig. (**4A**, **B**).

### 3.5 Higher-frequency of NK cells, Th/memory, CTLs and Treg cells, but Reduced Total T Cells were Observed in HV-PRRSV Infected as Compared with NADC-20 Infected Pigs

The frequency of various lymphocyte populations after infection was monitored by flow cytometry. In blood, the frequency of total T cells and NK cells in HV-PRRSV infected pigs were significantly higher than the NADC-20 infected pigs Fig. (**5A**). In contrast, HV-PRRSV infection significantly decreased Th/memory cell population in blood samples of pigs as compared with NADC-20 infected pigs. There were no significant differences among the groups for all other cell populations assayed. The frequency of Th/memory, CTLs, and T-reg cells in the lung of HV-PRRSV infected pigs were significantly higher than that in NADC-20 infected pigs Fig. (**5B**). However, the total T cell population in HV-PRRSV infected pigs was significantly lower than NADC-20 infected pigs. There was no difference for the percentage of T-helper cells and γδ T cells in the lung between the two infected groups.

## 4 DISCUSSION

The HP-PRRSV has been reported to induce high fever, loss of body weight, severe respiratory symptoms and high mortality. In our study, body temperature in HV-PRRSV infected pigs was higher than 40°C during the duration of the infection, which may partially contribute to dehydration and respiratory distress Fig. (**1A**). The HV-PRRSV infection led to significant pig body weight loss as compared with NADC-20 infected pigs. The HV-PRRSV infected pigs lost an average of 10% of their body weight at 3 and 6 DPI, however, body weight returned to original weight by 10 DPI Fig. (**1C**). The body weight of NADC-20 infected pigs increased consistently after infection, although it was significantly lower when compared with the control pigs at 6 and 10 DPI (Fig. **1C**). Consistent with a previous report [14, 15], HV-PRRSV infected pigs also showed more severe clinical symptoms including cutaneous hemorrhages and cyanotic extremities on the edge of ears (“blue ear”) and higher mortality rate (3/5 pigs died).

HV-PRRSV led to significant thymus atrophy compared with NADC-20 infection. The ratio of thymus/total body weight was significantly lower in HV-PRRSV infected pigs as compared with NADC-20 infected pigs (Fig. **1C**). Thymus is the primary lymphoid tissue, in which T-lymphocytes mature and constitute the peripheral T-cell repertoire responsible for directing many facets of the adaptive immune responses. The malfunction/atrophy of thymus leads to the depletion of T lymphocytes, which was consistent with the significant loss of total T lymphocytes in the lung analyzed by flow cytometry. In contrast, the ratio of lung/total body weight was significantly higher in HV-PRRSV infected pigs as compared with NADC-20 infected pigs (Fig. **2**), which indicated more inflammatory responses after HV-PRRSV infection. Several T cell subpopulations which exert cytotoxic functions, such as CTLs (CD3^+^CD4^-^CD8^+^) and Th/memory (CD3^+^CD4^+^CD8^+^), were significantly higher after HV-PRRSV infection when compared with NADC-20 infection.

It has been reported that HP-PRRSV has higher proliferation ability than classic PRRSV both *in vitro* and *in vivo* [5]. In this study, both HV-PRRSV and NADC-20 showed similar proliferation ability within the first 6 DPI. Interestingly, by 9 DPI the viremia in NADC-20 infected pigs declined while the viremia of HV-PRRSV infected pigs was still increasing (Fig. **3A**). In a study by Guo *et al*. [5], the virus titer and virus load in the serum were significantly higher after rJXwn6 HP-PRRSV infection as compared with VR-2332 infection from 2 to 11 DPI. The discrepancy of the viremia level could be due to the different strains of PRRSV used in each study, and the NADC-20 used in our study is more virulent than VR-2332. However, the high proliferation ability of HV-PRRSV did not correlate with higher titer of PRRSV-specific IDEXX ELISA antibody response, in that the average antibody titer in NADC-20 infected pigs was significantly higher than HV-PRRSV infected pigs at 9 DPI (Fig. **3B**). The IDEXX ELISA measures the antibody response against N proteins of PRRSV, which has no protective ability to the PRRSV infection although it has been widely used for field diagnosis [16]. The different abilities to induce PRRSV IDEXX ELISA Ab between HP-PRRSV and classic PRRSV may contribute to the pathogenesis of viruses, and need further exploration.

TNF-α is a pro-inflammatory cytokine, which plays a very important role in regulation of immune responses, fever development (inflammation), and cell apoptosis [17]. Several studies showed that PRRSV down-regulated TNF-α production in the early stage of infection, which may be used by virus to circumvent infected cell apoptosis [18, 19]. At the late stage of PRRSV infection, the peak of both apoptotic cells and viral antigen expression were observed in lymph nodes and tonsils of infected animals [20]. In our study, HV-PRRSV induced significantly higher TNF-α in both serum and lung samples at 6 DPI, and the high level of TNF-α expression correlates with the high level of viremia. The coincidence between high expression of TNF-α and high level of viremia at the late stage of PRRSV infection may indicate that PRRSV induces TNF-α mediated cell apoptosis to release virion progeny to infect other vulnerable cells.

Previous studies have shown that infection with several classic strains of PRRSV virus induced delayed or failed production of detectable serum IFN-α [21-23]. In contrast, HV-PRRSV infection induced significantly higher IFN-α in the serum of pigs but significantly lower levels in the lung samples. Working as a potent antiviral molecule, IFN-α was reported to significantly inhibit PRRSV replication and enhance cellular-mediated immunity (IFN-γ responses) [24, 25]. However, the elevated serum IFN-α has no effect on virus clearance by the evidence of high level of viremia in HV-PRRSV infected pigs at 9 DPI (Figs. **3A** and **4A**). Also, the low level of IFN-α expression in the lung tissue after HV-PRRSV infection did not lead to decreased IFN-γ production as compared with NADC-20 infected pigs. Therefore, the role of IFN-α in the pathogenesis of PRRSV and host immunity to combat PRRSV needs to be further explored.

HV-PRRSV also elicited a significant elevation of adaptive immunity cytokines in the serum samples, such as IL-10 and IFN-γ, and significantly higher IL-10 in the lung samples (Figs. **4A, B**). Induction of IL-10 following PRRSV infection is believed to be a focal mechanism leading to the unique immunological outcomes and interference of PRRSV vaccine efficacy. The production of IL-10 in the early stage of PRRSV infection is associated with a wide array of PRRSV-induced immunomodulatory activities [24, 26]. Consistent with previous studies, the expression of IL-10 in the serum and lung samples was significantly higher in HV-PRRSV infected pigs when compared with NADC-20 infected pigs [5]. The high level expression of IL-10 correlates with high titer of viremia in this study and PRRSV antigen gene expression in the lungs and tonsils of PRRSV infected pigs in previous studies [22]. Some strains of modified live PRRSV vaccines also induced IL-10 production in vaccinated pigs, which may partially contribute to the failure of PRRSV vaccination [24]. Therefore, circumventing the inhibitory effect of IL-10 in the early stage of PRRVS vaccination/infection could be a challenge for PRRSV vaccine development.

**Fig. (5):**
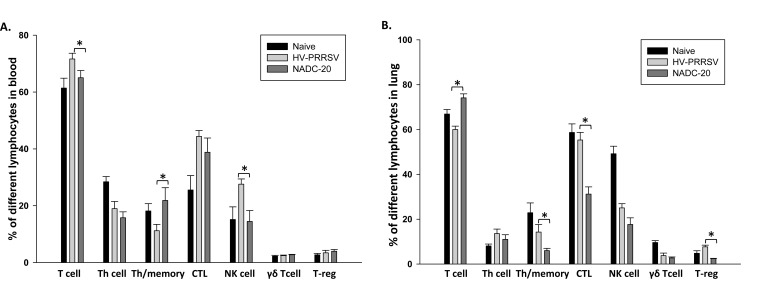
**T lymphocyte population profiles after PRRSV infection.** Whole blood **(A)** and lung samples **(B)** were collected at necropsy and were used to analyze the percentage of different T lymphocyte populations by flow cytometer according to their phenotypes. Data were shown as mean ± SEM for 5 pigs per group. One asterisk denotes a statistically significant difference (P <0.05), and “NS” denotes there was no statistically significant difference.

IFN-γ is a key cytokine associated with host cell-mediated immunity (CMI) response, and is secreted by natural killer cells and several different T cell subpopulations. Significantly higher levels of IFN-γ in the serum was found in pigs infected with HV-PRRSV as compared with NADC-20 infected pigs (Fig. **4A**), which was associated with a significantly higher percentage of NK cells in the blood (Fig. **5A**). The coincidence of high levels of IFN-γ expression and the high percentage of NK cells may indicate that the production of IFN-γ at this stage might be a result of the innate immune response, most likely from antigen-stimulated NK cells [27]. However, the higher level of IFN-γ in the serum did not lead to lower level of viremia in these pigs. The level of viremia in HV-PRRSV infected pigs was significantly higher than that in NADC-20 infected pigs. Therefore, the role IFN-γ plays in the protection to PRRSV infection at this stage is questionable.

## CONCLUSION

In conclusion, by comparing immune responses in pigs infected with two virulent PRRSV strains: Chinese HV-PRRSV and U.S. NADC-20 strain, we found that the high proliferation ability of HV-PRRSV did not enhance the early production of PRRSV-specific ELISA antibodies (Ab). HV-PRRSV induced higher levels of TNF-α and IL-10 in both serum and the lung and higher levels of IFN-α and IFN-γ in the serum than did NADC-20. Flow cytometry analysis showed that HV-PRRSV infected pigs generated significantly higher frequencies of NK cells in the peripheral blood and Th/memory, CTLs, and T-reg cells in the lung tissue as compared with NADC-20 infected pigs. Thus, this study demonstrates that different immunity profiles were elicited by HV-PRRSV and NADC-20, and these differences may contribute to the better understanding of the pathogenesis of HP-PRRSV.
